# Clinical Significance of Nadir Hemoglobin in Predicting Neurologic Outcome in Infants With Abused Head Trauma

**DOI:** 10.3389/fped.2020.00140

**Published:** 2020-04-03

**Authors:** En-Pei Lee, Lu-Lu Zhao, Shao-Hsuan Hsia, Jung Lee, Oi-Wa Chan, Chia-Ying Lin, Ya-Ting Su, Jainn-Jim Lin, Han-Ping Wu

**Affiliations:** ^1^Division of Pediatric Critical Care Medicine, Department of Pediatrics, Chang Gung Memorial Hospital at Linko, Taoyuan, Taiwan; ^2^College of Medicine, Chang Gung University, Taoyuan, Taiwan; ^3^Department of Pediatrics, Taipei Tzu Chi Hospital, New Taipei, Taiwan; ^4^Department of Medicine, School of Medicine, Tzu Chi University, Hualien, Taiwan; ^5^Division of Pediatric General Medicine, Department of Pediatrics, Chang Gung Memorial Hospital at Linko, Taoyuan, Taiwan; ^6^Department of Pediatric Emergency Medicine, Children's Hospital, China Medical University, Taichung, Taiwan; ^7^Department of Medical Research, Children's Hospital, China Medical University, Taichung, Taiwan; ^8^Department of Medicine, School of Medicine, China Medical University, Taichung, Taiwan

**Keywords:** brain injury, trauma, intracranial hemorrhage, nadir, hemoglobin, infant

## Abstract

Traumatic brain injury (TBI) is a leading cause of pediatric morbidity and mortality and is categorized as abusive head trauma (AHT) and accidental head injury. A retrospective chart review of 124 children aged <1 year diagnosed with TBI were analyzed. Outcomes were evaluated at discharge and 6 months later by using the Pediatric Cerebral Performance Category (PCPC) Scale. The receiver operating characteristic (ROC) curve was applied to determine the cutoff values for hemoglobin (HB) levels. In the study, 50 infants (40.3%) achieved a favorable neurologic outcome (PCPC ≦ 2) and 74 (59.7%) had poor neurologic outcomes (PCPC ≧ 3). Infants with poor neurologic outcomes had lower HB on admission and nadir HB (*p* < 0.05). Based on multivariate logistic regression analysis, the nadir HB was a predictor of poor neurologic outcomes at discharge and 6 months later in both AHT and accidental head injury. Nadir HB had the largest area under the ROC curve for predicting poor neurologic outcomes. We determined the appropriate cutoff value of nadir HB as 9.35 g/dl for predicting neurologic outcomes in infants with TBI. Furthermore, the cutoff value of nadir HB in predicting poor neurologic outcomes in infants caused by AHT and accidental head injury were taken as 9.36 and 8.75 g/dl, respectively.

## Introduction

Physical abuse and neglect are the major types of child maltreatment and affect an increasing number of children in the world (1). Despite the efforts of child protection medical services, physical abuse and neglect still remain important and serious problems. Based on the data of the National Child Abuse and Neglect Data System (NCANDS) in the United States of America, the number of fatalities fluctuated in the past 5 years. Younger children are the most frequent victims of child maltreatment because of their dependency and inability to protect themselves. Children aged <3 years old accounted for about three-quarters of all fatalities, especially infants (<1-year-old) who accounted for half of all fatalities in the USA in 2015 due to child maltreatment (2). In the infant group suffering from child maltreatment, the primary cause of death is traumatic brain injury (TBI). In general TBI can be classified as abusive head trauma (AHT) and accidental head injury (3–5). Intracranial hemorrhage is frequent after TBI in infants due to the highly vascularized nature of the brain and is associated with poor outcomes. About two thirds of AHT and quarter of accidental head injury have the feature of subdural hemorrhage (SDH). And epidural hemorrhages (EDH) were significantly associated with accidental head injury rather than AHT (17 vs. 4 percent, respectively) (6). Therefore, it is important to analyze the risk factors associated with morbidity and mortality in infants with traumatic intracranial hemorrhage to develop guidelines for adequate treatment to prevent further complications such as higher brain dysfunction.

In adults with intracranial hemorrhage caused by TBI or stroke, several risk factors such as the volume of intracranial hemorrhage, the score on the Glasgow Coma Scale (GCS) on admission, or older age are predictors of poor outcomes. In particular, the common laboratory examination of HB can be used as a predictor for outcome, with low HB indicating poor functional outcomes after non-traumatic brain hemorrhage at discharge and at 3 months (7, 8). The pathophysiology of anemia in TBI or stroke is evidence of primary blood loss. The severe impact of anemia on brain function in various pathological conditions is somewhat unclear. It may be that lower HB impaired the function of brain oxygenation (9). However, evidence about the utility of HB in pediatric intracranial hemorrhage is limited, and its use is not endorsed in predicting outcomes of pediatric intracranial hemorrhage. The main objective of this study was to determine whether the HB level in traumatic intracranial hemorrhagic infants is associated with poor neurologic outcomes. Our hypothesis was that low HB is associated with increased mortality or poor neurologic outcomes.

## Methods

### Study Design

From January 2006 to 2017, we retrospectively collected data from infants aged <1 years old with intracranial hemorrhage (ICH) caused by TBI who were admitted to the pediatric intensive care unit (PICU) via the emergency department (ED) or outpatient department (OPD). A total of 124 infants with ICH confirmed by brain computed tomography (CT) at admission were included in this study. The study was approved by the Institutional Review Board of the Chang Gung Memorial Hospital. All methods were performed in accordance with the relevant guidelines and regulations.

The data were collected, reviewed, de-identified, and anonymously analyzed by the authors, and the Ethics Committee waived the requirement for informed consent because of the anonymized nature of the data, and scientific purpose of the study.

### Study Setting and Patient Selection

The setting in our study was a tertiary medical center receiving cases transferred from local clinics, regional hospitals, and social and politic institutions (child protection agency subordinated by the government). The PICU of our hospital was a tertiary ICU with 29 beds for hospitalized patients aged <18 years. In our study, all the victims with ICH caused by child maltreatment by the definition of World Health Organization (WHO) (10) were admitted to the PICU. Victims with accompanying internal bleeding from other organs (except intracranial bleeding) were excluded. After review of the clinical history, physical examination findings, and past medical charts, TBI was categorized as AHT and accidental head injury according to predefined criteria (Table 1) (3–5). All the victims of AHT and accidental head injury were viewed by the child protection team. Our hospital was a Child Protection Medical Service Demonstration Center which consisting of well-trained medical doctors, nursing staff, and social workers for identifying abused and neglected victims.

**Table 1:** Classification scheme of head injuries as abuse or accident.

**AHT:**
a. Witnessed inflicted head injury
b. Confession of abuse by perpetrator
c. History of traumatic event which isn't compatible with injury severity
d. Physical injuries consistent only with inflicted injuries (eg, old and new lesions, pattern bruises)
e. No history resulting in patient's head injury
**Accidental Head Injury:**
a. History of traumatic event which is compatible with injury severity
b. Witnessed accident (eg, falls, sports-related injuries, isolated or unique event)
c. Absence of physical injuries which are consistent only with inflicted injuries (eg, old and new lesions, pattern bruises)
d. Vehicle accidents

*AHT, abusive head trauma*.

### Study Protocol

Information related to the cases of AHT and accidental head injury, including age (months), gender, initial presentations, Glasgow Coma Score (GCS), injury severity score (ISS), imaging examinations, length of stay in hospitals, and ICUs, laboratory data such as hemoglobin (HB) levels, neurologic outcomes, and mortality were obtained from the social welfare reporting system and medical records. Admission of victims to the PICU may indicate an urgent clinical condition requiring critical care following the guidelines of the Society of Critical Care Medicine (11), meeting at least one of its defined criteria. Rotterdam scoring system was used in the study to grade acute TBI on the basis of CT findings. It included four independently scored elements such as degree of basal cistern compression, degree of midline shift, traumatic subarachnoid, or intraventricular hemorrhage, and epidural hematoma (12). Neurologic outcome at discharge was evaluated by pediatric neurologists using the system of Pediatric Cerebral Performance Category (PCPC) Scale. Neurologic outcomes at 6 months after discharge were evaluated by pediatric neurologists in the OPD, using the PCPC scale. Outcome scores were divided into favorable (PCPC 1–2) and poor neurologic outcomes (PCPC 3–6 at discharge; PCPC 3–5, 6 months later). The patients with favorable neurologic outcomes (PCPC 1–2) could go to the regular school, whereas poor neurologic outcomes could only go to the special school or respiratory care ward.

### Exposure

The exposures of concern were admission HB, nadir HB (the lowest HB value during hospital stay), and mean HB (calculated from all values within 1 month). Daily or more frequent rechecks of HB were performed if the initial HB was <9 g/dl or in the event of any hypotension episode. All infants with a nadir of 9 g/dl or less received red blood cell (RBC) transfusions.

### Statistical Analysis

The chi-square test, Fisher's exact test, Student's *t*-test, Mann–Whitney *U*-test, and multivariate logistic regression analysis were used where appropriate. In the descriptive analysis, values are presented as mean ± standard deviation (SD) or median (interquartile range). The difference between groups is presented as 95% confidence intervals (CIs). For comparison of dichotomous variables between groups, the chi-square test or Fisher's exact test was used. Comparisons of continuous variables between two groups were made with the Mann–Whitney *U*-test. Predicted probabilities of a poor neurologic outcome (PCPC scale 3–6 at discharge and PCPC scale 3–5 at 6 months) and 95% confidence intervals were calculated by using a logistic regression model.

Finally, the receiver operating characteristic (ROC) curve was applied to determine the ideal cut-off values of HB for poor neurologic outcomes. The test characteristics of the different cut-off values, including sensitivity, specificity, area under the curve (AUC), positive likelihood ratio (LR^+^), and negative likelihood ratio (LR^−^) were also examined. Statistical significance was defined at the *p* < 0.05 level and all statistical analyses were conducted using IBM SPSS Statistics software (version 22.0; SPSS Inc., Chicago, IL, USA).

## Results

### Participant Characteristics

In the study, among the 124 infants, fifty (40.3%) infants achieved a favorable neurologic outcome and 74 (59.7%) had poor neurologic outcomes. The comparison of good neurologic outcomes and poor neurologic outcomes in both groups are listed Table 2. In the cohort, 78 (62.9%) were classified as suffering from AHT and 46 (37.1%) were classified as suffering from accidental head injury. At initial presentation, only the initial GCS score was significantly lower in infants with poor neurologic outcomes in both groups (*p* < 0.05). The brain image findings showed no significant difference between good and poor neurologic outcomes in both groups. But the Rotterdam CT score were significant higher in the poor neurologic group in both group (*p* < 0.05). The admission HB and nadir HB were both significantly lower but the numbers for RBC transfusion were significantly higher in the poor neurologic group (all *p* < 0.05).

**Table 2:** Correlation between poor outcome and characteristics in the group with AHT and accidental head injury.

**Characteristics**	**AHT (*****n*** **=** **78)**		***P*-value**	**Accidental head injury (*****n*** **=** **46)**		***P*-value**
	**PCPC 1–2**	**PCPC 3–6**		**PCPC 1–2**	**PCPC 3–6**	
Patients, *n* (%)	12 (15.3)	66 (84.7)		38 (82.6)	8 (17.4)	
Age month, mean (SD)	5.9 ± 4.3	5.1 ± 3.3	0.63	6.4 ± 3.7	8.1 ± 4.3	0.18
Sex (male, *n*, %)	9 (75)	31 (46.9)	0.14	21 (55.2)	4 (50)	0.92
LOS in ICU, median (IQR)	4 (3–11)	13 (7–23)	0.1	4 (3–7)	9 (6–11)	0.024^*^
Mortality, *n* (%)	0	8 (12.1)		0	1 (12.5)	
Initial presentation						
Initial GCS, median (IQR)	14 (11–15)	10 (6–13)	0.029	15 (15–15)	8 (6–9)	<0.01^*^
Initial ISS, mean (SD)	21.5 ± 4.7	30.3 ± 18.3	0.16	15.3 ± 3.3	23.4 ± 20.9	0.15
Hypotension, *n* (%)	0 (0)	14 (21.2)	0.17	0 (0)	2 (25)	0.028^*^
Retinal hemorrhage, *n* (%)	7 (58.3)	45 (68.1)	0.74	1 (2.6)	2 (25)	0.12
Brain CT findings, *n* (%)						
SDH	9 (75)	47 (71.2)	0.92	18 (47.3)	7 (87.5)	0.09
EDH	1 (8.3)	0	0.33	13 (34.2)	3 (37.5)	0.8
SAH	2 (16.6)	22 (33.3)	0.41	6 (15.7)	3 (37.5)	0.35
IVH	0	7 (10.6)	0.52	1 (2.6)	1 (12.5)	0.77
DAI	2 (1.6)	14 (21.2)	1	0	1 (12.5)	0.38
Skull Fracture, *n* (%)	1 (8.3)	8 (12.1)	0.92	16 (42.1)	2 (25)	0.61
Rotterdam CT score, median (IQR)	2 (2–3)	3 (2–4)	0.01^*^	2 (2–2)	3 (2–3)	0.02^*^
2	9 (75)	22 (33.3)		35 (92.1)	2 (25)	
3	3 (25)	25 (37.8)		3 (7.9)	5 (62.5)	
4	0	14 (21.3)		0	1 (12.5)	
5	0	5 (7.6)		0	0	
Laboratory findings, median (IQR)						
Admission Hb g/dL)	10.3 (9.5–11.3)	9 (7.6–9.9)	0.028^*^	10.7 (9.4–12.1)	8.5 (7.6–8.8)	<0.01^*^
Mean Hb (g/dL)	10.4 (9.5–11)	10.2 (9.3–10.8)	0.33	10.9 (10.2–12)	8.7 (8.6–9.5)	<0.01^*^
Nadir Hb (g/dL)	10.3 (9–10.4)	8.1 (7.3–9.1)	<0.01^*^	10.4 (9.2–11.9)	7.2 (6.3–7.6)	<0.01^*^
Acute Neurosurgical Interventions, *n* (%)	8 (66.6)	43 (65.1)	0.823	11 (28.9)	6 (87.5)	0.01^*^
Burr hole drainage	7 (58.3)	39 (59.1)	0.96	5 (13.1)	3 (37.5)	0.25
External ventricular drainage	1 (8.3)	3 (4.5)	0.58	1 (2.6)	1 (12.5)	0.77
Intracranial pressure monitor	0	11 (16.6)	0.12	0	1 (12.5)	0.38
Craniotomy and hematoma evacuation	2 (16.6)	4 (6.1)	0.49	5 (13.1)	2 (25)	0.76
Decompressive craniectomy	0	1 (1.5)	0.66	0	0	−
Treatment, *n* (%)						
Mechanical ventilation	4 (33.3)	40 (60.6)	0.158	2 (5.9)	4 (50)	<0.01^*^
RBC transfusion	3 (25)	47 (71.2)	0.002^*^	5 (13.2)	6 (75)	<0.01^*^

(*): %;

* *p < 0.05 statistic significant; AHT, abusive head trauma; ICU, intensive care unit; LOS, length of stay; GCS, glasgow coma scale; ISS, injury severity score; SDH, subdural hemorrhage; EDH, epidural hemorrhage; SAH, subarachnoid Hemorrhage; IVH, intraventricular Hemorrhage; DAI, diffuse axonal injury; Hb, Hemoglobin*.

### Association Between HB Levels, Clinical Risk Factors, and Poor Outcomes

**Figure 1:**
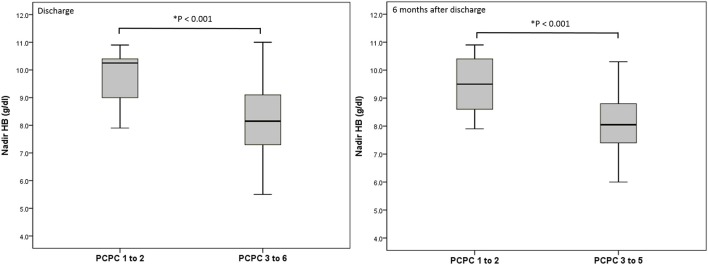
Nadir HB (g/dl) and outcome at discharge and 6 months after discharge in the group with AHT.

**Figure 2:**
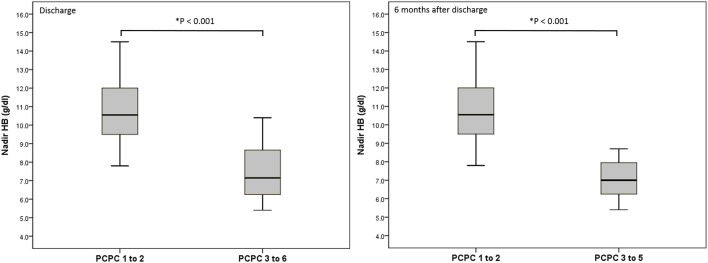
Nadir HB (g/dl) and outcome at discharge and 6 months after discharge in the group with accidental head injury.

We performed a logistic regression model to identify indicators for predicting poor neurologic outcome at discharge and 6 months after discharge, and found that initial GCS score, ISS, nadir HB and Rotterdam CT score were all significant factors (Table 3). In the final model, only nadir HB remained as an independent predictor of poor neurologic outcome at discharge, but nadir HB and the initial GCS score were predictors of poor neurologic outcome at 6 months after discharge in the abused group. In the group with accidental head injury, only nadir HB appeared as an independent predictor for poor neurologic outcome at 6 months after discharge. And the detected mean time of nadir HB was 3.6 ± 3.5 h after admission. In-hospital mortality was 9.7% (12 infants) (Table 4). Based on the results of univariate analysis, the initial GCS score, nadir HB and Rotterdam CT score were associated with in-hospital mortality. In the multivariate logistic regression model, only the initial GCS score (OR 0.528, 95% CI 0.389–0.716, *p* < 0.001) remained an independent predictor for in-hospital mortality (Table 5). Outcome at 6 months was available for 112 (90.3%) infants; and 12 infants died during admission. The nadir HB levels showed significant differences between groups with favorable and poor neurologic outcomes in the group with AHT (Figure 1) and accidental head injury (Figure 2) at discharge and 6 months after discharge (both *p* < 0.05).

**Table 3:** Multivariate Logistic regression model to predict poor neurologic outcome (PCPC ≧ 3) at discharge and 6 months.

**Parameters**	**Discharge**		**6 months after discharge**	
	**Adjusted Odds ratio**	***p*-value**	**Adjusted Odds ratio**	***P*-value**
AHT				
Initial GCS	0.910 (0.721–1.126)	0.361	0.806 (0.653–0.994)	0.044^*^
Injury severity score	1.058 (0.908–1.232)	0.47	1.042 (0.877–1.238)	0.642
Nadir HB (g/dl)	0.332 (0.156–0.707)	0.004^*^	0.381 (0.205–0.709)	0.002^*^
Rotterdam CT score	2.351 (0.872–7.351)	0.13	1.691 (0.639–4.475)	0.29
Accidental head injury				
Initial GCS	0.532 (0.249–1.138)	0.104	0.558 (0.257–1.211)	0.14
Injury severity score	1.122 (0.869–1.448)	0.376	0.883 (0.288–2.703)	0.827
Nadir HB (g/dl)	0.078 (0.007–0.884)	0.039^*^	0.083 (0.008–0.874)	0.038^*^
Rotterdam CT score	22.13 (0.063–77.29)	0.3	20.143 (0.349–81.42)	0.147

The adjusted odds ratios were obtained from a multivariate logistic model which included sex, initial GCS, ISS, image findings, retinal hemorrhage, neurosurgery, skull bone fracture, nadir HB;

* *p < 0.05 statistic significant; AHT, abusive head trauma; GCS, Glasgow Coma Scale; ISS, injury severity score; HB, hemoglobin*.

**Table 4:** Univariate analysis of factors associated with in-hospital mortality.

**Variables**	**Survivor**	**Mortality**	***P*-value**
Patient number	112	12	
Age (month)	6 ± 3.6	4.2 ± 3.2	0.069
Sex (male, *n*, %)	72 (63.7)	6 (54.5)	0.78
Initial GCS	12.2 ± 3.2	4.7 ± 2.6	<0.01^*^
Admission HB (g/dl)	9.6 (8.7–10.8)	9.6 (6.4–10.4)	0.338
Mean HB (g/dl)	10.2 (9.5–11.1)	10.3 (9.3–11)	0.577
Nadir HB (g/dl)	8.8 (7.9–10.3)	7.3 (6.3–8.5)	0.014^*^
RBC transfusion (*n*, %)	53 (46.9)	8 (72.7)	0.187
Rotterdam CT score	2 (2–3)	4 (3–5)	<0.01^*^

(*): %;

* *p < 0.05*.

*GCS, Glasgow Coma Scale; HB, hemoglobin*.

**Table 5:** Logistic regression model to predict mortality.

**Logistic regression**	**Odds ratio**	***P*-value**
Univariate		
Initial GCS	0.528 (0.389–0.716)	<0.001
Nadir HB (g/dl)	0.6 (0.415–0.867)	0.007
Rotterdam CT score	4.493 (2.102–9.603)	<0.01
Multivariate		
Initial GCS	0.528 (0.389–0.716)	<0.001

(*): %; * p < 0.05 statistic significant.

*GCS, Glasgow Coma Scale; HB, hemoglobin*.

**Figure 3:**
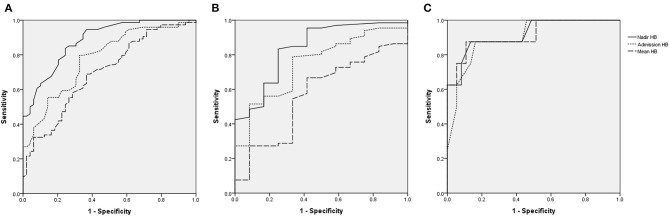
Receiving operating characteristic (ROC) curves for assessing the predictive accuracy of HB for poor functional outcomes. **(A)**, All victims. **(B)**, AHT. **(C)**, Accidental head injury.

Nadir HB had the highest area under the ROC curve (AUC) for predicting poor neurologic outcomes in all victims (Figure 3A). When divided into group with AHT (Figure 3B) and accidental head injury (Figure 3C), the nadir HB also had the largest AUC for predicting poor neurologic outcomes in each group. The AUCs of nadir HB, mean HB, and admission HB for predicting good or poor neurologic outcome in the all victims, victims with AHT and accidental head injury were calculated. The AUC of nadir HB was 0.884 (95% CI, 0.827–0.941) in the all group; 0.839 (95% CI, 0.718–0.96) in the group with AHT; and 0.919 (95% CI, 0.808–1) in the group with accidental head injury which were all significantly higher than both admission HB and mean HB in all groups (all *p* < 0.001) (Table 6). The best cutoff values of nadir HB in predicting poor neurologic outcomes are shown in Table 7. We identified nadir HB levels of 9.35 g/dl in the cohort, 9.36 g/dl in the group with AHT and 8.75 g/dl in the group with accidental head injury to predict poor neurologic outcomes at discharge.

**Table 6:** The victims and ROC analysis at the parameters of HB between good and poor neurologic outcome.

**All victims *N* = 124**	**AUC**	**AHT *N* = 78**	**AUC**	**Accidental head injury *N* = 46**	**AUC**
Nadir HB (g/dL)	0.884	Nadir HB (g/dL)	0.839	Nadir HB (g/dL)	0.919
Admission HB (g/dL)	0.778	Admission HB (g/dL)	0.747	Admission HB (g/dL)	0.9
Mean HB (g/dL)	0.699	Mean HB (g/dL)	0.569	Mean HB (g/dL)	0.916

*AHT, abusive head trauma; AUC, area under receiver operating characteristic curve*.

**Table 7:** Best predictive power of nadir HB for different group at discharge.

**Group**	**Nadir HB (g/dl) (AUC)**	**Sensitivity**	**Specificity**	**LR^**+**^**	**LR^**−**^**	**Youden index**
All (*n* = 124)	9.35 (0.884)	0.838	0.755	3.42	0.21	0.59
AHT (*n* = 78)	9.36 (0.839)	0.833	0.75	3.33	0.22	0.58
Accidental (*n* = 46)	8.75 (0.919)	0.875	0.86	6.47	0.14	0.73

*AHT, abusive head trauma; LR^+^, positive likelihood ratio; LR^−^, Negative likelihood ratio*.

In addition, we compared RBC transfused and non-transfused patients in Table 8 which reporting that the RBC transfused group had more critical initial presentations and worse outcomes including lower initial GCS, higher ISS, more episodes of hypotension, lower admission HB, nadir HB, and higher percentage of poor neurologic outcomes.

**Table 8:** Comparison between RBC transfused and non-transfused patients.

**Characteristics**	**Non-transfused**	**RBC transfused**	***P*-value**
Patients, *n* (%)	63	61	
Age month, mean (SD)	6.7 ± 3.4	5 ± 3.6	0.008
Sex (male, *n*, %)	41 (65.1)	37 (60.6)	0.711
LOS in ICU, median (IQR)	9.2 ± 12.5	18.7 ± 21.3	0.003^*^
Initial presentation			
Initial GCS, median (IQR)	15 (13–15)	10 (7–13)	<0.001^*^
Initial ISS, mean (SD)	21.7 ± 11.1	29.2 ± 18.3	0.003^*^
Hypotension, n (%)	2 (3.1)	14 (22.9)	<0.001^*^
Retinal hemorrhage, n (%)	26 (41.2)	44 (72.1)	0.001^*^
Brain CT findings, *n* (%)			
SDH	47 (74.6)	54 (88.5)	0.064
EDH	12 (19)	5 (8.1)	0.116
SAH	18 (28.5)	22 (36.1)	0.445
IVH	5 (7.9)	11 (18)	0.113
DAI	8 (12.6)	14 (22.9)	0.162
Skull Fracture, *n* (%)	15 (23.8)	14 (22.9)	0.91
Laboratory findings, median (IQR)			
Admission Hb (g/dL)	10.4 (9.5–11.7)	8.7 (7.4–9.6)	<0.001^*^
Mean Hb (g/dL)	10.5 (9.8–11.4)	10 (9.2–10.6)	0.725
Nadir Hb (g/dL)	10.2 (9.3–10.9)	7.6 (6.8–8.3)	<0.001^*^
Treatment, *n* (%)			
Neurosurgical interventions	26 (41.2)	46 (75.4)	<0.001^*^
Mechanical ventilation	12 (19)	38 (62.2)	<0.001^*^
Outcomes, *n* (%)			
PCPC 1 to 2	42 (66.6)	8 (13.1)	<0.001^*^
PCPC 3 to 6	21 (33.4)	53 (86.8)	
Mortality, *n* (%)	3 (4.7)	8 (13.1)	0.123

(*): %;

* *p < 0.05 statistic significant; RBC, red blood cell; ICU, intensive care unit; LOS, length of stay; GCS, glasgow coma scale; ISS, injury severity score; SDH, subdural hemorrhage; EDH, epidural hemorrhage; SAH, subarachnoid Hemorrhage; IVH, intraventricular Hemorrhage; DAI, diffuse axonal injury; Hb, Hemoglobin*.

## Discussion

Low HB is a common complication in patients with intracranial hemorrhage (13). In adults with ICH, low HB levels and initial levels of consciousness may be associated with poor functional outcome (7, 14, 15). Initial GCS scores related to poor functional outcome have been reported in some studies (16, 17), but few related studies focused on this field in pediatric cases. We believe that our study is the first to analyze and report the role of nadir HB in predicting neurologic outcomes in infants with traumatic ICH, and to identify how low HB levels may be used as a predictor for poor neurologic outcome in infants. In this study, we found that nadir HB was a more powerful predictor for predicting poor neurologic outcomes at discharge and 6 months after discharge than both the initial GCS score and other forms of HB. The pathophysiology of anemia-related poor neurologic outcomes in patients with ICH is mainly caused by two factors: first, anemia can reflect the volume of primary blood loss, which is a powerful predictor for poor prognosis (9, 18, 19); second, anemia may cause secondary brain damage resulting from cerebral hypoxia. The cerebral oxygen delivery (DO_2_) is equal to cerebral blood flow (CBF) multiplied by arterial oxygen content (CaO_2_). More oxygenation is needed in the acute phase of an injured brain, but impaired global cerebrovascular autoregulation develops in patients with ICH causing compensatory mechanisms to fail (9, 13, 20). The results indicate that although the CBF increases in patients with an injured brain, it is still insufficient to conquer the reduced CaO_2_ induced by the low HB. Consequently, decreased levels of cerebral DO_2_ develop and result in hypoxic damage to the brain.

Previous studies have demonstrated that admission HB, mean HB, and nadir HB are all the predictors for poor prognosis in adults with ICH (7, 8, 21, 22), but they did not report which one was the most useful. In our study, according to the results of the final logistic mode, nadir HB appeared to be the best predictor of poor neurologic outcomes in infants with ICH. We hypothesize nadir HB may reflect the proportion of primary blood loss and the lowest CaO_2_ level indicating the severity of cerebral hypoxia. Therefore, once nadir HB develops within the 1st day after admission, it may indicate that nadir HB could play a feasible and practical role in predicting poor neurologic outcome. However, in infants with traumatic brain injury, we noticed that no related studies mentioned the issue in detail, so we determined the cutoff value of nadir HB as 9.35 g/dl to predict poor neurologic outcomes in infants with ICH, based on the ROC analysis, which may provide an indicator for primary clinicians to perform early interventions for infants with ICH. Moreover, we determined the cutoff value of nadir HB in predicting poor neurologic outcomes in infants with traumatic ICH caused by AHT and accidental head injury as 9.36 and 8.75 g/dl, respectively.

In previous studies, the initial GCS score and ISS were identified as risk factors for poor prognosis in pediatric cases, (23, 24). However, in our retrospective study, we found that nadir HB, initial GCS, and ISS were all related factors, and nadir HB may be more effective than the initial GCS score and ISS for predicting poor prognosis. The present study found that all of the nadir HB were detected within 12 h after admission (3.6 ± 3.5 h). Therefore, the nadir HB also provided other information: if the initial brain CT and admission HB were not critical to indicate aggressive surgical intervention, the lower nadir HB could be a parameter for monitoring disease progression and the need for further surgical intervention. In our study, we surveyed the related factors for in-hospital mortality, and found that the initial GCS score remained the independent predictor for in-hospital mortality in the multivariate mode rather than nadir HB. This may be because 66.6% (8 of 12) of mortalities presented with out-of-hospital cardiac arrest and a GCS score of 3. Considering the brain lesions, 91.7% (11 of 12) of mortalities had diffuse axonal injuries with hypoxia and hypotension, a potential risk for in-hospital mortality (25). In consequence, initial GCS scores are a powerful predictive value for in-hospital mortality in infants with traumatic ICH.

In our study, we also found why accidental head injury and AHT showed different cutoff value of nadir HB in predicting outcomes and different blood transfusion strategies. We think that the reason may be due to different trauma mechanisms. As we know, the trauma mechanisms are quietly different between the group with accidental head injury and AHT. In the group with accidental head injury, EDH resulting from injured meningeal artery is more common causing by one trauma episode. Repeated shaking head at different time points resulting in acute on chronic SDH, diffuse axonal injury may be common in the group with AHT. EDH may cause more blood loss than SDH potentially. The different blood transfusion strategies and different outcomes may be based on the different trauma mechanisms.

Until now, there is no golden standard for blood transfusion in adult and pediatric TBI (26, 27). Previous studies demonstrated that HB lower than 7 g/dl will cause anemia-induced brain injury. But the optimal transfusion threshold is still controversial. The transfusion threshold from 7 to 10 g/dl is the common transfusion strategy in the clinical practice. There is still no conclusion whether blood transfusion is associated with better or worse outcomes. Some studies demonstrated RBCT is benefit (28, 29) or harmful (30, 31) or no impact (32, 33) for neurologic outcome in patients with TBI. Our multivariate logistic regression analysis was not powered to detect an association between RBC transfusion and poor prognosis. Further randomized controlled trials based on different transfusion thresholds settings are required.

## Conclusions

We demonstrated the association between nadir HB and poor neurologic outcome in infants with traumatic ICH. We determined the best cutoff value of nadir HB to be 9.35 g/dl for predicting poor neurologic outcomes in infants with traumatic ICH. Furthermore, the cutoff value of nadir HB in predicting poor neurologic outcomes in infants with ICH caused by AHT and accidental head injury were taken as 9.36 and 8.75 g/dl, respectively.

## Data Availability Statement

All datasets generated for this study are included in the article/supplementary material.

## Ethics Statement

The studies involving human participants were reviewed and approved by the Institution Review Board and ethics committee of Chang-Gung Memorial hospital. IRB No.: 104-8307B. Written informed consent to participate in this study was provided by the participants' legal guardian/next of kin.

## Author Contributions

E-PL, L-LZ, and S-HH conceived and designed the study. L-LZ and S-HH participated in data analysis. O-WC and C-YL gathered the data. E-PL, Y-TS, and JL drafted the manuscript. H-PW and J-JL designed and oversaw the study, interpreted the data. H-PW revised the manuscript. All authors have read and approved the final manuscript for publication.

### Conflict of Interest

The authors declare that the research was conducted in the absence of any commercial or financial relationships that could be construed as a potential conflict of interest.
